# Advances in Drug Therapy for Metastatic Pancreatic Ductal Adenocarcinoma

**DOI:** 10.7150/jca.89788

**Published:** 2024-02-25

**Authors:** Jianjun Gao, Jiangang Wang, Canghai Guan, Wujiang Shi, Qingfu Dong, Jialin Sheng, Xinlei Zou, Zhaoqiang Xu, Yifei Ge, Ziyue Huang, Jiehan Li, Haolin Bao, Yi Xu, Yunfu Cui, Xiaoxue Xu, Xiangyu Zhong

**Affiliations:** 1 Department of Hepatopancreatobiliary Surgery, The Second Affiliated Hospital of Harbin Medical University, Harbin 150086, Heilongjiang, China.; 2 Department of General Surgery, Tangdu Hospital, Air Force Medical University, Xi'an 710032, Shanxi, China.; 3 Key Laboratory of Basic Pharmacology of Ministry of Education, Zunyi Medical University, Zunyi, 563006, Guizhou, China.; 4 Key Laboratory of Functional and Clinical Translational Medicine, Fujian Province University, Xiamen Medical College, Xiamen, 361000, Fujian, China.; 5 State Key Laboratory of Chemical Oncogenomics, Key Laboratory of Chemical Genomics, Peking University Shenzhen Graduate School, Shenzhen, 518055, Guangzhou, China.; 6 Jiangsu Province Engineering Research Center of Tumor Targeted Nano Diagnostic and Therapeutic Materials, Yancheng Teachers University, Yancheng, 224007, Jiangsu, China.; 7 Key Laboratory of Biomarkers and In Vitro Diagnosis Translation of Zhejiang province, Hangzhou,310000, Zhejiang, China.; 8 Key Laboratory of Gastrointestinal Cancer, Fujian Medical University, Ministry of Education, School of Basic Medical Sciences, Fujian Medical University, Fuzhou, 350122, Fujian, China.; 9 School of Health Administration, Harbin Medical University, Harbin 150086, Heilongjiang, China.

**Keywords:** metastatic pancreatic cancer, metabolic pathways, DNA damage systems, tumor microenvironment, immune system

## Abstract

Pancreatic ductal adenocarcinoma (PDAC) is an aggressive disease with a notably poor prognosis. A large number of patients with PDAC develop metastases before they are diagnosed with metastatic pancreatic cancer (mPDAC). For mPDAC, FOLFIRINOX or gemcitabine plus nab-paclitaxel are the current first-line treatments. It is important to note, however, that many patients will fail chemotherapy because of drug resistance. ​Heterogeneous tumors and complex tumor microenvironments are key factors. As a result, clinical researchers are exploring a variety of alternative treatment modalities. Current understanding of the molecular signature and immune landscape of PDAC has motivated the emergence of different targeted and immune-based therapeutic approaches, some of which have shown promising results. The purpose of this review is to discuss the new targets and new drugs for mPDAC in terms of specific pathogenic factors such as metabolic vulnerability, DNA damage repair system, tumor microenvironment and immune system, in order to identify potential vulnerabilities in mPDAC patients and hopefully improve the prognosis of mPDAC patients.

## 1. Introduction

There is an overall five-year survival rate of only 11% for pancreatic ductal adenocarcinoma (PDAC), which is rather low compared with many other cancer types 1. Currently, radical surgical resection and systemic palliative chemotherapy are the main treatment strategies for patients with PDAC. Among them, patients with early diagnosis of tumor diameter less than 3 cm have a relatively good prognosis, and the 5-year survival rate after surgery can reach 40% 2. But the recurrence rate of these patients remains high, with approximately 70% relapsing within two years of treatment 3. Approximately 50% of patients are diagnosed with progressive pancreatic cancer at an early stage or develop metastases to distant organs that cannot be treated with radical surgery and can only be treated with systemic palliative chemotherapy 4. The main metastatic sites of PDAC are liver, lung, lymph node, peritoneal and adrenal metastases, of which about 70% of PDAC patients develop liver metastases during their disease 5. Data show that the median life expectancy for metastatic PDAC (mPDAC) is approximately 1 year, with a 5-year OS rate of less than 2% 6.

**Figure 1:**
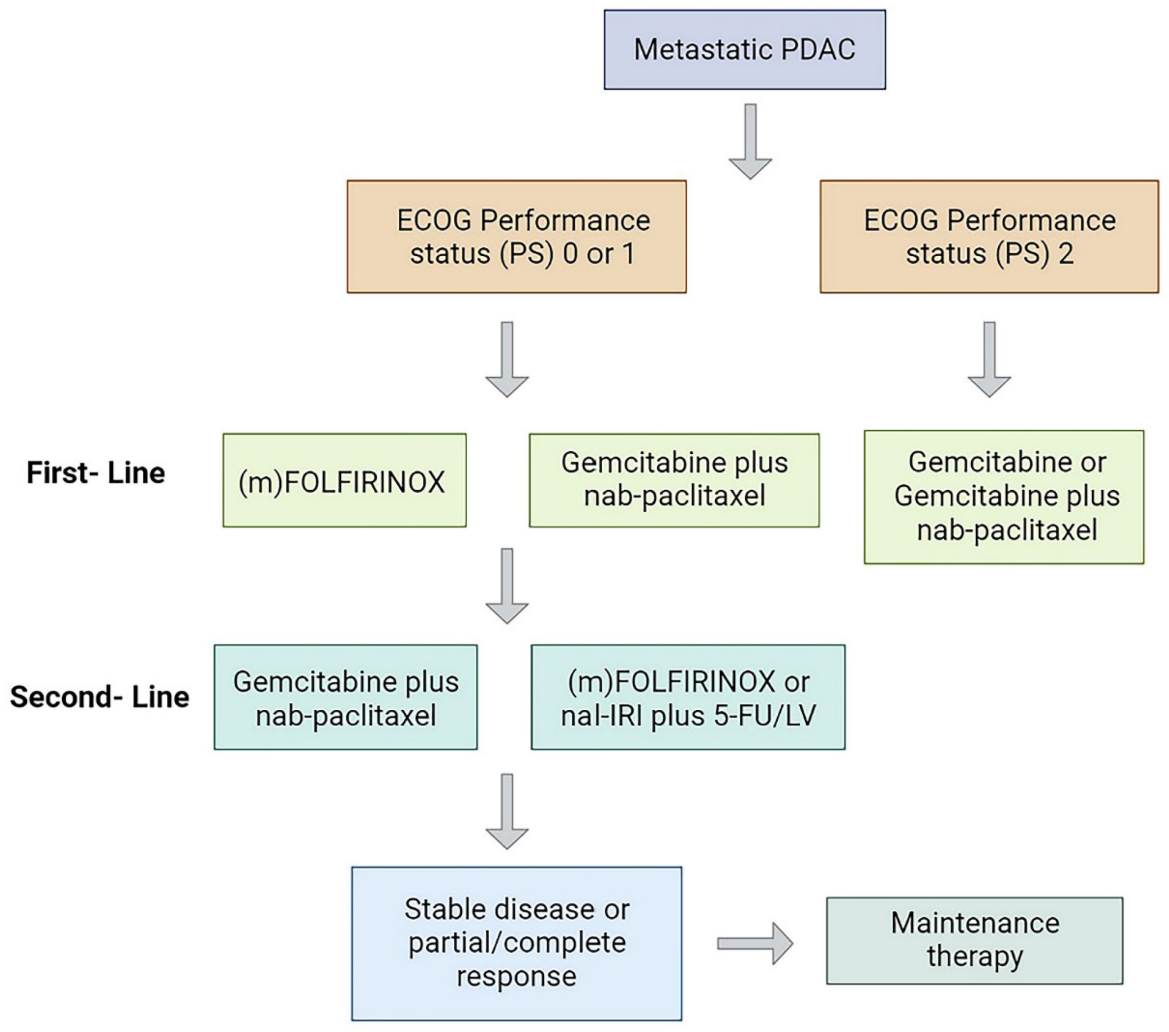
Treatment strategy in patients with mPDAC. PS, performance status; mFOLFIRINOX, modified FOLFIRINOX.

Currently, the standard treatment modality for mPDAC is systemic palliative chemotherapy (Figure 1), and the first-line regimen includes gemcitabine plus nab-paclitaxel (GnP) (ECOG 0-2) or (modified) (m)FOLFIRINOX (ECOG 0-1) with median overall survival (mOS) of 8.5 and 11.1 months, respectively 7-9. Gemcitabine-based therapy is generally the second-line option for patients treated with FOLFIRINOX in the first line 10. In October 2015, the Food and Drug Administration (FDA) approved nanoliposomal irinotecan combined with fluorouracil and folinic as a second-line treatment for mPDAC, a regimen that prolonged survival in mPDAC patients previously received gemcitabine-based therapy with an mOS of 6.1 months vs 4.2 months with fluorouracil and folinic acid 11. Recently, a randomized phase III clinical trial showed that the PARP inhibitor olaparib could be used for maintenance therapy in patients with mPDAC and BRCA1/2 mutations (only 4% to 7% in pancreatic cancer patients) receiving platinum-based chemotherapy and prolonged progression-free survival (PFS) 12. Although these drugs have produced some efficacy in mPDAC, the long-term survival of patients have not improved significantly 13.

Mutations in genes and altered molecular pathways have become crucial targets for precision therapy of PDAC patients. Numerous researchers have developed strategies to target mutated genes in pancreatic cancer, such as KRAS, NRG1, and NTRK, but most results are unsatisfactory 14. The genes BRCA and ATM, which are involved in stabilizing the chromosome structure, are defective in some PDAC patients, making them potential therapeutic targets 15. In addition, therapies such as targeting metabolic pathways, targeting tumor microenvironment (TME) and immune checkpoint inhibition have also shown potential for targeting pancreatic cancer cells 16, 17 (Table 1). In this review, we highlight recent advances in preclinical studies and clinical trials for mPDAC patients.

## 2. Targeting metabolic vulnerabilities

Reprogramming of glucose, protein, lipid and nucleic acid metabolism provides tumor cells with energy and responds to redox stress 18. PDAC cells rely on aerobic glycolysis (Warburg effect) or metabolic changes to adapt to energy and nutrient deprivation and abnormal oxidative stress in the TME 19. Based on the metabolite profiles of PDAC cells, Daemen et al. 20 classified them into three subtypes: low proliferating, glycolytic, and lipogenic. Glycolysis and glutamine are sensitive to inhibitors of glycolysis. In contrast, lipoproteins are more sensitive to lipid inhibitors, but not to inhibition of the glycolytic pathway. Thus, a deeper understanding of the PDAC metabolic reprogramming process could help to develop better drugs for relevant targets.

### 2.1 Inhibition of TCA

**Figure 2:**
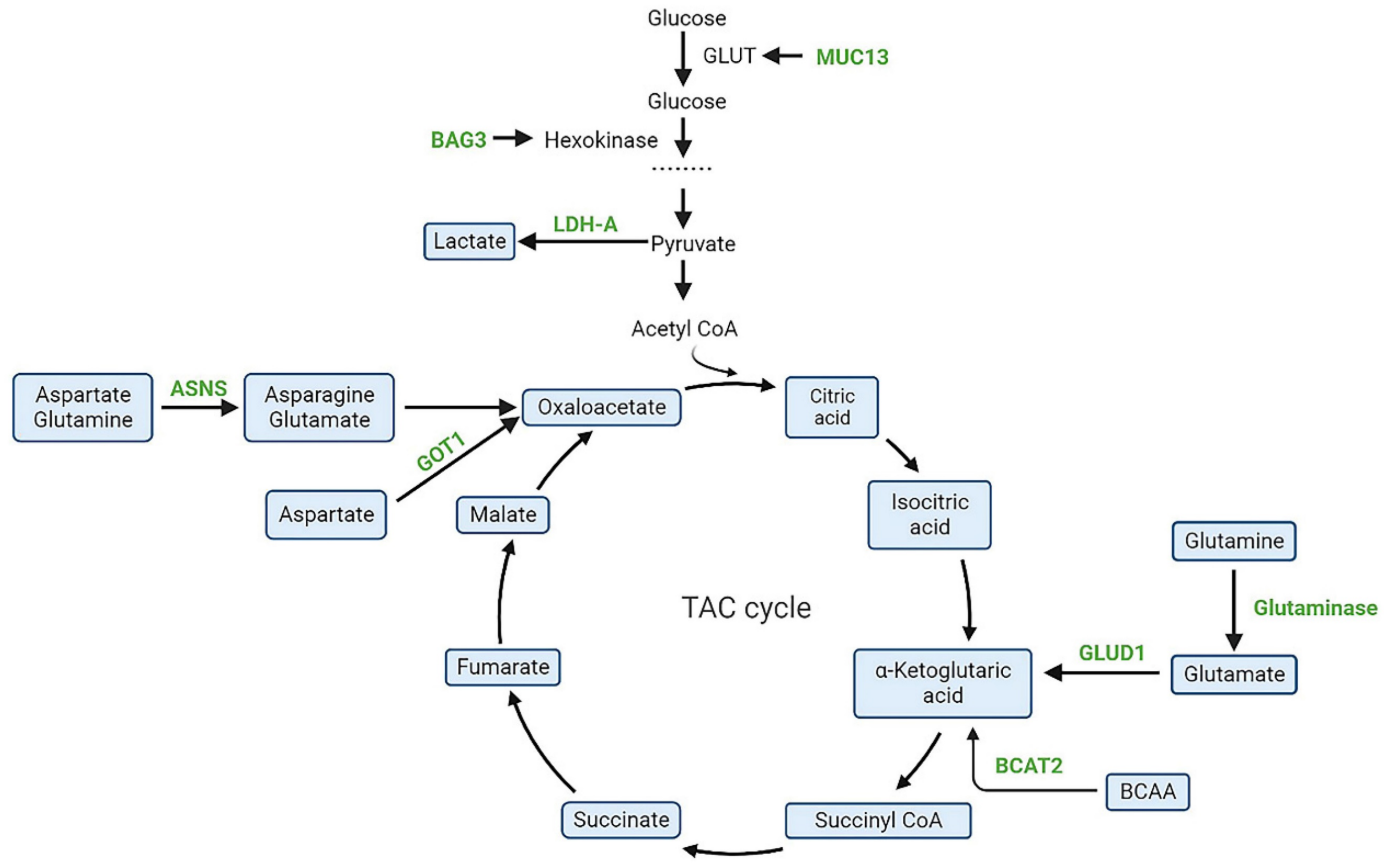
Schematic representation of targeted metabolic intermediates and enzymes(green) in PDAC. BAG3, Bcl-2-associated athanogene 3; MUC13, mucin 13; LDH-A, lactate dehydrogenase A; GOT1, aspartate aminotransferase; ASNS, asparagine synthetase; BCAT2, branched-chain amino acid transaminase 2; GLUT, glucose transporter; GLUD1, glutamate dehydrogenase-1.

A key pathway in the glycometabolism is glycolysis, which is the primary source of energy for the growth and proliferation of cells. Tricarboxylic acid cycle (TCA) cycle intermediates play an important role in the metabolic abnormalities of PDAC and may be potential therapeutic targets. Currently, molecules related to glycolytic pathways such as ME2/3 21, PON2 22, LDH-A 23, BAG3 24, MUC1/13 25, long noncoding RNA-PLACT1 [26]and IKKε 27 have been shown to play crucial roles in PDAC (Figure 2). However, more exploration is needed for drug development.Devimistat is a lipid analogue that selectively targets the TCA cycle in tumor cells and thus selectively acts on cancer cells. It inhibits pyruvate dehydrogenase and α-ketoglutarate dehydrogenase complexes to reduce the entry of glucose and glutamine derived carbons into the TCA cycle, ultimately leading to cancer cell death 28. In a phase I clinical study, 20 mPDAC patients treated with devimistat and mFOLFIRINOX in combination demonstrated better drug tolerance and a 61% objective response rate (ORR) 29. Subsequently, a global, randomized phase III trial evaluating devimistat in combination with mFOLFIRINOX versus FOLFIRINOX in patients with mPDAC yielded results with no significant improvement in ORR, PFS or OS with the combination (NCT03504423) 30. In addition, a single arm, open-label, phase I trial evaluated the combination of devimisat with GnP in mPDAC patients and showed that devimisat can be safely administered with GnP and nab-paclitaxel at doses up to 1500 m/g2 31.

### 2.2 Inhibition of amino acids metabolism

Studies have shown that glutamine plays an essential role in the growth and proliferation of PDAC cells 32. Son et al. showed that PDAC cells maintain an intracellular redox state through a non-classical glutamine metabolic pathway in which glutamate-derived aspartate is converted to oxaloacetate, malate, and pyruvate by aspartate transaminase (GOT1). However, in normal cells, glutamate dehydrogenase (GLUD1) can convert glutamine-derived glutamate to α-ketoglutarate in order to enter the TCA cycle 33. Similarly, Abrego et al. 32 showed that under low PH conditions, PDAC cells deviated from glycolytic metabolism and relied more on oxidative metabolism promoted by increased GOT1 enzyme expression. Zhou et al. 34 suggested that GOT1 plays an important role in coordinating glycolysis and oxidative phosphorylation pathways in KRAS mutated cancer cells. Since GOT1 plays a key role in abnormal glutamine metabolism, it may be a potential anti-cancer target. Wu et al. 35 found that the cardiovascular drug hydrazine hydrochloride can inhibit the catalytic activity of GOT1, but its mechanism needs to be further investigated. The potential of asparagine as a target for PDAC treatment has been discovered. Most cells are capable of transforming asparagine and glutamine into asparagine and glutamate through asparagine synthetase (ASNS). In stark opposition, l-Asparaginase, a chemotherapeutic agent, hydrolyzes asparagine to aspartic acid and ammonia, thus depriving cells of the circulating asparagine and thus influencing the proliferation and growth of cancer cells. Studies have shown that PDAC is a tumor with low ASNS expression, and therefore, patients may benefit from l-Asparaginase treatment 36, 37. A randomized IIb open-label study revealed that the combination of erythrocyte-encapsulated l-ASNase (eryaspase) and gemcitabine/mFOLFOX-6 as second-line therapy enhanced both overall survival and progression-free survival (NCT02195180) 38, however, independent of ASNS expression. In a randomized, open-label phase III trial (Trybeca-1), the combination of eryaspase with GnP or irinotecan/5-FU failed to meet the primary endpoint of patient OS, however, in the irinotecan/5-FU subgroup, eryaspase group showed good tolerability and survival benefit and warrants additional study (NCT03665441) 39. Another phase I trial used eryaspase in combination with mFOLFIRINOX for the first-line treatment of locally advanced or mPDAC (NCT04292743), with results to be further observed 40. In addition, Pathria et al. 41 found that inhibition of asparagine activated receptor tyrosine kinase-MAPK signaling and enhanced translation of transcription factor 4 (ATF4) mRNA in pancreatic cancer cells. Similarly, inhibition of the MAPK pathway reduces ATF4 translation and ASNS expression, increasing the sensitivity of pancreatic tumors to asparagine restriction 42. ​Thus, the combination of inhibition of the MAPK pathway and Asparagine may be a new therapeutic measure for PDAC patients with low ASNS expression. Racemetyrosine (SM-88) is a dysfunctional tyrosine derivative that causes disruption of protein synthesis in malignant tumor cells. MUC1 is a key target for this process 43. Cancer cells display heightened levels of free radicals or reactive oxygen species (ROS). SM-88 controls ROS levels by interfering with tumor cell MUC1 activity, leading to increased toxicity and ultimately triggering apoptotic signaling and cell death 44. In a prospective, open-label randomized phase II/III clinical trial in metastatic pancreatic cancer, SM-88 in combination with MPS (methoxsalen, phenytoin and sirolimus) was found to be well tolerated, with an overall patient disease control rate (DCR) of 27% (NCT03512756) 45. DCR is the percentage of patients with advanced tumors who result in a complete response, partial response, or stable disease through a therapeutic intervention. In addition, a multi-center, phase III trial enrolling 825 patients with metastatic pancreatic cancer is currently enrolling (NCT04229004) to evaluate the efficacy of pamrevlumab, a monoclonal antibody to SM-88 or anti-connective tissue growth factor (CTGF), compared to first-line chemotherapy agents GnP or mFOLFIRINOX. Semi-essential amino acid arginine promotes nitric oxide production and protein synthesis in cells 46. In pancreatic cancer, the rate-limiting enzyme for arginine synthesis, the enzyme argininosuccinate synthetase (ASS1), is commonly absent 47. Arginine deiminase converts arginine to citrulline and prevents arginine production in cells 48. ​Thus, arginine deiminase could be a novel therapeutic approach for PDAC. Preclinical study found that arginine depletion using pegylated arginine deiminase (ADI-PEG20) selectively inhibited the proliferation of ASS1-deficient pancreatic cancer cells and enhanced radiotherapy-induced apoptosis 49. Moreover, ADI-PEG20 increased the sensitivity of pancreatic cancer to gemcitabine 50. A single-arm, non-randomized, open-label, phase I/Ib trial showed that the combination of ADI-PEG20, gemcitabine, and nab-paclitaxel was well-tolerated in patients with advanced pancreatic cancer 51. The potential for ADI-PEG20 and other drugs to augment the activity and anti-tumor capacity of T cells could be a significant factor in the future treatment of advanced pancreatic cancer 52, 53.

### 2.3 Regulating the autophagy

Autophagy is a process that maintains metabolism and cellular homeostasis through the lysosomal breakdown of intracellular organelles and macromolecules. Pancreatic cancer exhibits elevated autophagy under basal conditions, and inhibition of autophagy leads to elevated DNA damage, increased ROS, decreased mitochondrial oxidative phosphorylation, and inhibition of PDAC cell growth 54. A randomized phase II study showed that preoperative addition of the autophagy inhibitor hydroxychloroquine (HCQ) to GnP chemotherapy improved surgical prognosis in patients with PDAC 55. The primary endpoint of OS at 1 year was not met in an open-label, phase II randomized clinical trial involving the combination of HCQ and GnP for mPDAC. However, the overall remission rates with HCQ were improved, indicating a potential role for HCQ in the locally advanced PDAC 56. A phase II study (NCT04669197) is treating locally advanced PDAC with a combination of paclitaxel protein bound + gemcitabine + cisplatin + HCQ and remains to be further evaluated. Preclinical studies have found that KRAS-mutant PDAC cells can activate autophagy to resist the inhibitory effects of MEK. Two studies are currently evaluating the efficacy of HCQ in combination with binimetinib or trametinib in patients with KRAS-mutant advanced or mPDAC (NCT04132505 and NCT03825289) 57, 58. In addition, the ERK inhibitor temuterkib has shown promise in BRAF- and KRAS-mutant pancreatic cancer models, tested alone or in combination with HCQ (NCT04386057), and paricalcitol and HCQ or in combination with GnP (NCT04524702) in advanced or mPDAC patients.

## 3. Targeting DNA damage repair system

**Figure 3:**
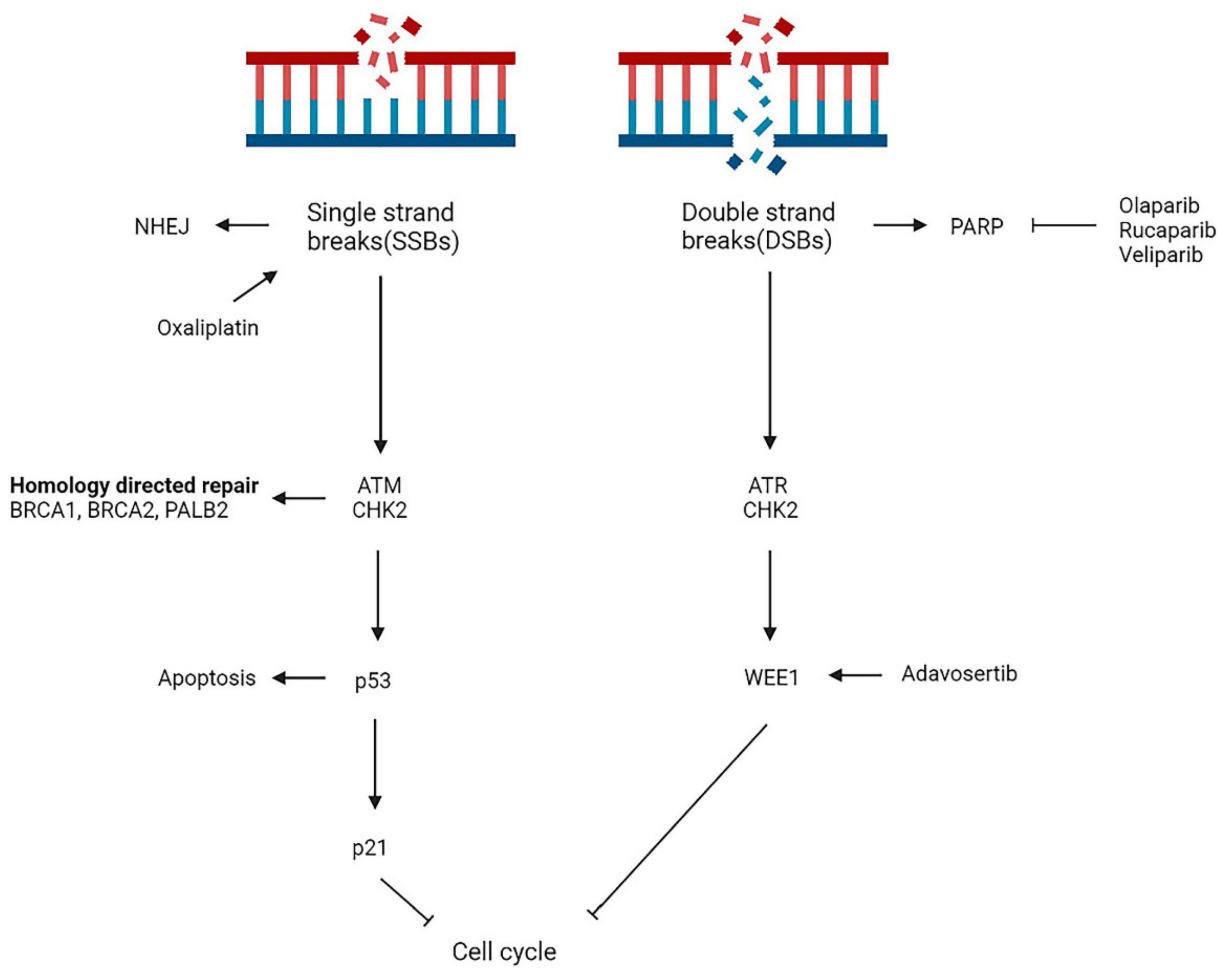
Overview of main DNA damage repair pathways in mPDAC. NHEJ, non-homologous end joining; PARP, poly ADP-ribose polymerase.

The damage or alteration of DNA molecules is a common phenomenon in cell division and, if not effectively repaired, can result in heritable genetic mutations. DNA damage activates the DNA damage repair (DDR) pathway to restore the structure of damaged DNA molecules, which is essential for maintaining genome stability 59. Damage to DDR at both endogenous (ROS) and exogenous (ionizing radiation or chemotherapies) factors can result in impaired DNA repair capacity and lead to malignant cellular transformation. Studies have shown that mutations in genes such as BRCA1, BRCA2, PALB2 and ATM cause homologous repair deficiency (HRD), with HRD reaching 14.5-16.5% in patients with PDAC 60, 61.HRD affects DNA double-strand break repair and increases sensitivity to poly ADP-ribose polymerase (PARP) inhibitors. PARP's involvement in DNA single-strand break (SSB) repair is of great significance, and its inhibition results in impaired repair capability and ultimately, the formation of DNA double-strand break (DSB) 62 (Figure 3).

### 3.1 PARP inhibitors

In a phase II clinical study, oralaparib, a PARP inhibitor, was initially investigated in patients with advanced cancer and BRCA 1/2 mutations. Among the 23 mPDAC patients, the ORR of olaparib was 21.7% and 35% of mPDAC patients had stable disease (SD) lasting ≥ 8 weeks 63. ​​The POLO trial validated the efficacy of olaparib in mPDAC patients, with a marked enhancement in PFS when compared to placebo, yet the OS remained comparable in both groups 64. On December 27, 2019, the FDA, having been presented with the evidence from the phase III randomized controlled trial, granted accelerated approval for olaparib to be used as a maintenance treatment for pancreatic cancer patients who have a germline BRCA mutation and have not progressed after 16 weeks of platinum therapy. A phase II clinical trial of olaparib for advanced PDAC patients with DDR deficiency without BRCA mutations (BRCAness) phenotype is underway, with preliminary results indicating an ORR of 20% and mPFS of 24.7 weeks 65. In addition, a phase II trial (NCT04548752) is evaluating olaparib and pembrolizumab as maintenance therapy for patients with BRCA mutations 66. Combination therapy with olaparib and the MEK inhibitor cobimetinib is being tested in mPDAC patients (NCT04005690) 67. The combination of cediranib maleate, an inhibitor of VEGFR tyrosine kinases, and olaparib in patients with solid tumors, including mPDAC (NCT02498613), is currently underway 68.

An investigation of the efficacy and safety of the oral PARP inhibitor olaparib in 19 patients with locally advanced/mPDAC and germline or somatic BRCA1/2 mutations was conducted in a single-arm, phase II RUCAPANC study. Rucaparib demonstrated clinical efficacy with a safety profile. ORR was 15.8% and DCR was 31.6% 69.

​In a single-arm, phase II clinical study, the PARP inhibitor veliparib was well-tolerated in phase III/IV PDAC in patients with BRCA1/2 or PALB2 mutations, but without tumor response 70. The combination of veliparib, oxaliplatin, 5-FU and calcium folinic acid (FOLFOX) is safe for those with mPDAC and has been demonstrated to be effective, particularly in those with BRCA1/2 or PALB2 mutations 71. In a phase I trial, De Bono et al. assessed the potency of talazoparib, a novel PARP inhibitor, in patients with advanced solid tumors and germline BRCA1/2 mutations. Single agent Talazoparib showed antitumor activity and tolerable safety 72.

### 3.2 ATR or WEE1 inhibitors

​ATMs are a key component of the DNA damage response, and they play a central role in activating the response to SSBs and DSBs 73. ​Disruption of ATM signals leads cells to rely on downstream ATR and CHK1/2 pathways to repair DNA, thus making them two potential targets for ATM-deficient tumors. In sporadic pancreatic cancer with somatic mutations, the overall mutation frequency of ATM is about 5% 74. Preclinical studies have found that ATM mutant PDAC cells are particularly sensitive to PARP, ATR and CHK1/2 inhibitors alone or in combination 75, 76. ATM-deficient tumors are currently being studied in clinical trials (NCT04514497 and NCT03682289). Studies show that WEE1 inhibition increases the effectiveness of DNA damaging agents and enhances the killing effect of combination drugs 77. A combination of a WEE1 inhibitor with a chemotherapeutic agent or radiation therapy has been evaluated and the results show promising results 78, 79. The ATM pathway and WEE1 may be crucial research directions for future studies of the DNA damage repair system in mPDAC patients. Routine germline and somatic cell testing, as recommended by the NCCN, should be conducted on all PDAC patients, thereby providing targeted treatment options for those with particular genomic alterations.

## 4. Targeting TME

**Figure 4:**
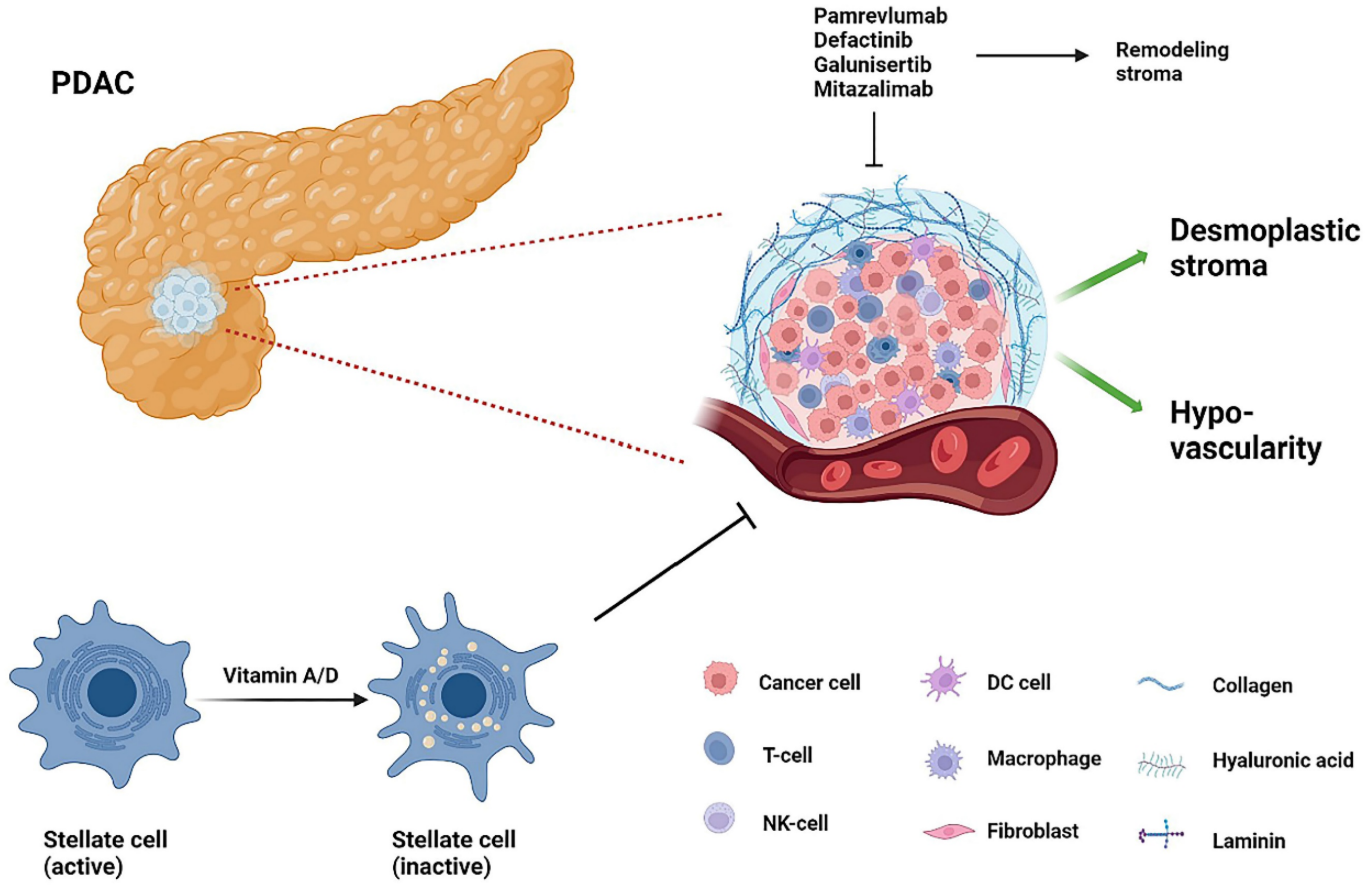
The tumor stroma of PDAC. T-cell, T-lymphocyte; NK-cell, natural killer cell; DC cell, dendritic cell.

PDAC's TME consists of a deeply pro-connective tissue proliferative and fibrotic mesenchyme that includes fibroblasts, stellate cells, endothelial cells, pericytes, neurons, infiltrating immune cells, and extracellular matrix proteins. This dense interstitium causes extreme interstitial fluid pressures, reduced vascularity and hypoxia, creating barriers to drug delivery to tumor cells, in which hyaluronic acid (HA) plays an essential role 80 (Figure 4). PEGPH20, a recombinant pegylated human hyaluronidase enzyme, is one of the most promising drugs. By reducing tumor pressure and vascular compression, PEGPH20 increases antitumor drug penetration and efficacy 80. However, a recent phase III clinical trial HALO109-301 combining PEGPH20 and GnP in previously untreated patients with hyaluronic acid-high stage IV mPDAC did not achieve the expected endpoints of OS and PFS and has been terminated 81.

MAESTRO, a phase III trial, revealed that Evofosfamide (TH-302), a selective hypoxia-activated precursor drug, could kill solid tumors with hypoxic microenvironments. Moreover, its combination with gemcitabine improved mPFS and OS in locally advanced or mPDAC, but the difference was not statistically significant 82. In addition, phase I clinical trials showed that the combination of TH-302 and ipilimumab did not show great efficacy in patients with mPDAC 83. ​Ibrutinib, a Bruton tyrosine kinase inhibitor with anti-mesenchymal fibrosis effects, failed to meet the primary endpoint of OS or PFS benefit when combined with GnP in patients with mPDAC 84. ​In addition, in patients with PDAC, ibrutinib had limited efficacy in combination with anti-PD-L1 antibodies developed and exported alone or in combination with pembrolizumab 85, 86. ​Despite the unsatisfactory results against mesenchymal-targeted drugs, the unique TME profile remains one of the hot spots for mPDAC therapy. ​Preliminary clinical trials have shown promising results, with final results pending further study 87. Immune cells are an integral part of TME and we will discuss immune system related treatments in the next subsection. This section will only discuss the rest of the TME mesenchyme in detail.

### 4.1 CTGF inhibitor

Pamrevlumab (FG-3019), a novel antifibrotic agent, has been found to be effective in suppressing the strong expression of CTGF in pancreatic cancer stroma 88. Early studies have shown that the combination of pamrevlumab and gemcitabine/erlotinib is well tolerated in locally advanced or mPDAC with no dose-limiting toxicity 89. In a phase I/II trial, the surgical resection rate for patients with locally advanced PDAC receiving GnP in combination with pamrevlumab was 33%, compared to 8% for GnP-only treatment 90. A phase III trial is evaluating effect of pamrevlumab in combination with GnP or FOLFIRINOX in patients with advanced PDAC, with results yet to be published (NCT03941093 and NCT04229004).​ Therefore, pamrevumab has the potential to alter poor outcomes in mPDAC patients.

### 4.2 FAK inhibitor

A non-receptor tyrosine kinase, focal adhesion kinase (FAK), is a major regulator of adhesion signaling and cell migration, and is commonly expressed in PDAC 91. ​It has been found that FAK inhibitors as monotherapy may inhibit tumor adhesion and migration, but do not have a significant effect on the prognosis of patients with advanced cancer, and that combination therapies may buffer the toxicity of other therapies that trigger cancer cell death or immune response 92. Preclinical data suggested that FAK inhibition synergizes with immune checkpoint inhibitors to increase the infiltration of cytotoxic T cells into tumors 93, 94. Using defactinib in conjunction with pembrolizumab and gemcitabine, Gillam et al. 95 published preliminary data on a FAK inhibitor combined with pembrolizumab. Among 20 patients with refractory PDAC, DCR was 80%, with one partial remission (PR) and 15 SDs, with mPFS and OS of 3.6 and 7.8 months, respectively. Of the 10 evaluable patients in the maintenance cohort, term response was 70%, with 1 PR and 6 SDs, and mPFS and OS were 5.0 and 8.3 months, respectively. The combination was well tolerated and safe, with good initial efficacy and demonstrated biomarker activity in infiltrating T lymphocytes. Additional clinical trials of defactinib plus pembrolizumab are currently being tested in resectable PC and advanced solid tumors (NCT03727880 96 and NCT02758587 97, respectively).

### 4.3 TGF-β inhibitor

Almost 50% of PDAC patients have mutations in SMAD4, a central component of TGF-β-mediated signalling 98. TGF-β promotes cell growth, epithelial to mesenchymal cell conversion, extracellular matrix remodelling and immunosuppression. Preclinical studies have shown that inhibition of the TGF-β pathway reduces tumor metastatic progression, fibroblast deposition and induces tumor cell apoptosis 99. Treatment of pancreatic cancer with galunisertib is being used as monotherapy or in combination with other drugs 100. Phase II studies of galunisertib in combination with gemcitabine demonstrated acceptable safety profiles (NCT01373164) 101. Galunisertib and gemcitabine improve OS and PFS in patients with pancreatic cancer, and patients with lower TGF-β levels may benefit more from galunisertib treatment 92. Melisi et al. 102 published preliminary results from a phase Ib study testing the efficacy of galunisertib and the anti-PD-L1 antibody durvalumab in mPDAC (NCT02734160). The combination of Galunisertib 150 mg twice daily and durvalumab 1500 mg Q4W was well-tolerated, with a DCR of 25%.

### 4.4 Vitamin analogues

Studies have demonstrated that the regulation of TME by vitamin D is a critical factor in the treatment of pancreatic cancer. Sherman et al. 103 have discovered that the mesenchymal stroma of pancreatic cancer expresses the vitamin D receptor (VDR). VDR, as a key regulator to promote the recovery of pancreatic stellate cells to the resting state, can be combined with gemcitabine to remodel mesenchyme and reduce tumor volume. Paricalcitol, a VDR activator, is currently in clinical trials and awaits publication 104.

All trans retinoic acid (ATRA), a derivative of vitamin A, can break down the dense stroma of pancreatic cancer and allow chemotherapeutic agents to reach the tumor site 105. Retinoic acid, in a mouse model of pancreatic cancer, induces quiescence of pancreatic stellate cells, reduces the Wnt/β-catenin signalling pathway, and induces apoptosis in the surrounding pancreatic cancer cells 106. A phase Ib dose escalation and expansion trial in patients with advanced, unresectable PDAC revealed that ATRA, when combined with GnP chemotherapy, was a safe and tolerable stromal-targeting agent (NCT03307148) 107. This combination will be further examined in a phase II randomised controlled trial in locally advanced PDAC (NCT04241276).

### 4.5 CD40 agonist

CD40, a member of the tumor necrosis factor receptor superfamily, is primarily expressed in leukocytes and some tumor cells. Studies have shown that CD40 activation prompts macrophages, but not T cells, to rapidly infiltrate tumors, become tumor-killing and promote tumor mesenchymal depletion 108. A multicentre phase Ib/II study, OTPIMIZE-1 (NCT04888312), was conducted to assess the safety, tolerability, and efficacy of mitazalimab (CD40 agonist) in conjunction with mFOLFIRINOX for the treatment of previously untreated mPDAC.The study is continuing and patients are currently being enrolled at a dose of 900µg/kg in combination with mFOLFIRINOX. In addition, a phase II randomised controlled trial investigated the use of GnP ± nivolumab ± sotigalimab. Antitumor activity was observed in all groups with a 1-year OS > 35%. A phase I trial is currently exploring the efficacy of the agonist CD40 monoclonal antibody CDX-1140 alone or in combination with CDX-301 (FLT3L), pembrolizumab or chemotherapy (NCT03329950).

## 5. Immunotherapy approaches

Immunotherapy and related immune checkpoint inhibitors (ICIs) such as programmed cell death protein 1 (PD-1), programmed cell death 1 ligand 1 (PD-L1) or cytotoxic T lymphocyte associated antigen-4 (CTLA-4) have produced positive results in the treatment of various solid tumors. Patients with PDAC respond poorly to immunotherapy due to low immunogenicity and low tumor mutation burden 109. In addition, the abundant mesenchyme produces a hypoxic microenvironment and activates cancer-associated fibroblasts and secretes TGF-β to drive the recruitment of immunosuppressive cells, thus hampering the role of immunotherapy 110. In spite of this, studies have found that a small percentage of PDAC patients still suffer from high microsatellite instability (MSI-h) or mismatch repair deficiency (dMMR) 111-113, and these patients, characterised by strong expression of tumor neoantigens and immune checkpoint ligands, have been shown to benefit from ICIs and to improve the rate of survival 114. The FDA has approved pembrolizumab, a checkpoint inhibitor of anti-PD-1, for MSI/dMMR tumors and is recommended by the NCCN as a second-line treatment 115. In addition, to overcome existing barriers to immunotherapy, researchers have used ICIs in combination with vaccines or cytotoxic therapies to boost immunogenicity in pancreatic cancer by recruiting and activating effector T cells that kill cancer cells.

### 5.1 Checkpoint inhibitors

The combination of the CTLA-4 immune checkpoint inhibitor ipilimumab and gemcitabine was safe and well-tolerated in PDAC patients, one of whom had a delayed response 116. A phase Ib, multisite, open-label, non-randomized trial was conducted to assess the safety and tolerability of tremlimumab in combination with gemcitabine in patients with mPDAC and warrants further study (NCT00556023) 117. ​A Ib/II single-center phase study investigating the anti-PD-1 antibody pembrolizumab in combination with GnP demonstrated a 100% DCR in 11 evaluable patients treated with mPDAC chemotherapy 118. Despite not achieving the primary goal of > 15% complete response, PFS and OS were superior to prior standard dose therapies for GnP. The combination of PD-1 inhibitor nivolumab with GnP has similarly shown great clinical benefit 119. However, the efficacy of GnP in combination with durvalumab and tremelimumab did not show a greater benefit in the randomised phase II trial (CCTG PA.7) (NCT02879318) 120. The practical use of ICIs in combination with additional drugs has also shown promise. An evaluation of the combination of ipilimumab and niraparib, a PARP inhibitor, as a maintenance therapy for advanced pancreatic cancer after 16 weeks of chemotherapy with stable outcomes was conducted in a phase I/II trial (NCT03404960) 121. 59.6% of patients did not progress at 6 months, compared with 44% for the pre-defined endpoint. Ongoing trials of immune checkpoint drug combinations in patients with mPDAC include durvalumab plus tremelimumab (NCT02558894) 122 and an evaluation of efficacy in combination with atezolizumab (NCT03193190) 123.

### 5.2 Therapeutic vaccines

**Figure 5:**
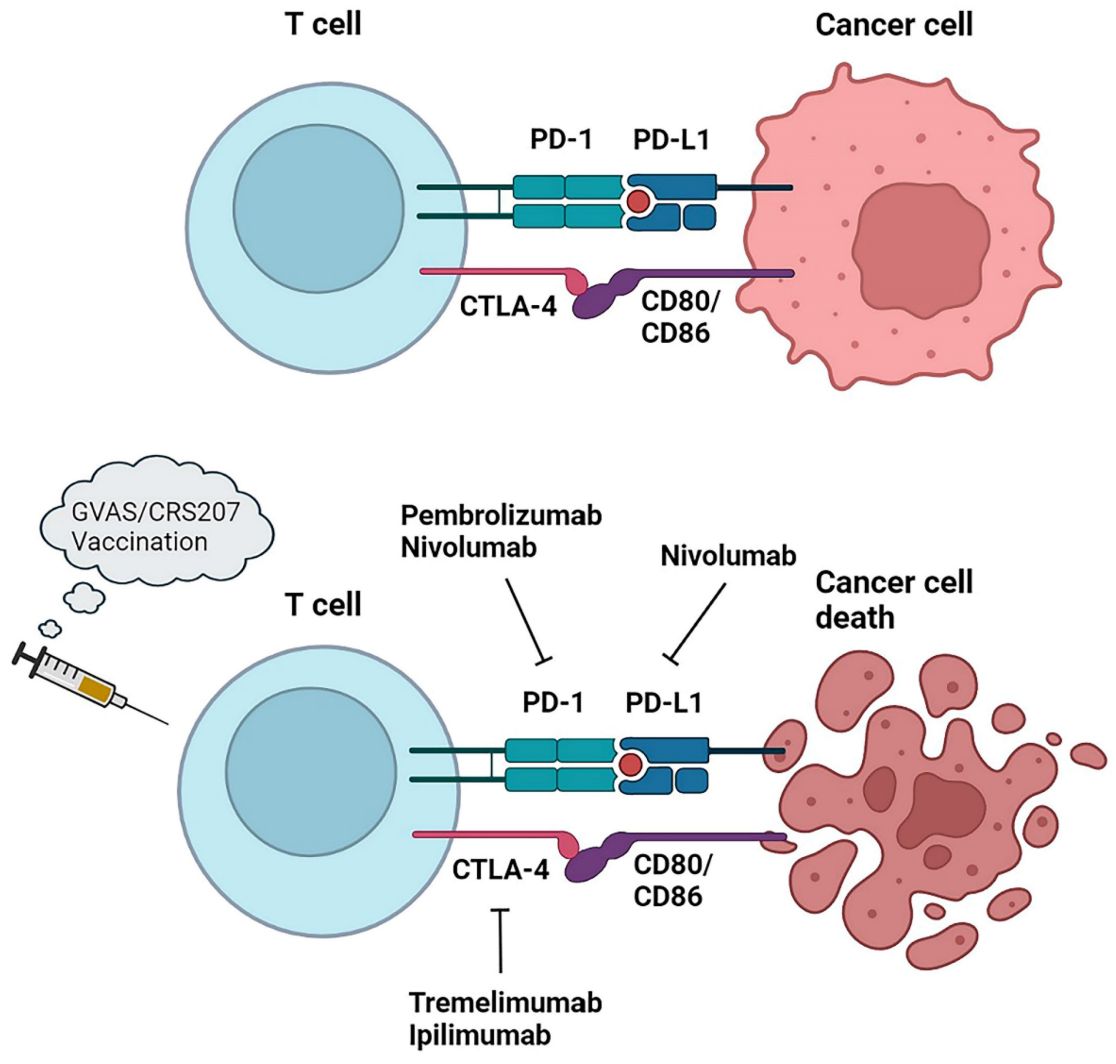
Immunotherapy approaches in PDAC. PD-1, programmed cell death protein 1; PD-L1, programmed cell death 1 ligand 1; CTLA-4, cytotoxic tlymphocyte associated antigen-4.

Cancer vaccines can achieve their killing effect by activating tumor-specific CD8+ T cells. A thick barrier in the TME of mPDAC hinders the infiltration of immune cells. By combining vaccine immunotherapy with therapies targeting TME, clinical regression in patients with mPDAC can be improved, such as by boosting T cell infiltration with hyaluronidase and augmenting immune cells with IL-2, IL-12, and TGF-β 124. On the other hand, the combination of a cancer vaccine with an adjuvant may also promote the immunogenicity of the vaccine and improve immunosuppressive and specific antigen deficiency in pancreatic cancer. Gene transfected tumor cell vaccine (GVAX), a whole cell vaccine expressing granulocyte-macrophage colony-stimulating factor (GM-CSF), is a gene transfected tumor cell vaccine. The cytokine GM-CSF plays an important role in DC antigen presentation. Combining GVAX with Listeria monocytogenes (Lm)-based vaccines can enhance DC function and enhance cellular immunity 125. CR-207 is a live attenuator that expresses mesothelin 126. Based on their study, Le et al. 127 indicated that CRS-207 significantly improved overall survival compared to GVAX alone in patients with mPDAC. A randomized, phase IIb, triple-arm trial was conducted to compare the combination of cyclophosphamide, GVAX and CRS-207, CRS-207 alone, or chemotherapy alone in patients with mPDAC who had been exposed to at least two cytotoxic agents, including gemcitabine, prior to the results. No significant differences in the primary OS endpoint was observed between the three groups (3.4 vs. 5.4 vs. 4.6 months). in the primary OS endpoint was observed between the three groups (3.4 vs. 5.4 vs. 4.6 months) 128. Combining cancer vaccines with ICIs can also enhance antitumor responses, such as in a phase II trial that combined GVAX with pembrolizumab, epacadostat, and cyclophosphamide, then add CRS-207 in a patient with metastatic pancreas cancer to test efficacy (NCT03006302) (Figure 5). Derived from human telomerase reverse transcriptase (hTERT), an enzyme overexpressed in PDAC, GV1001 is a peptide vaccine. A three-group, open-label, randomised phase III trial (TeloVac) evaluated GV1001 in combination with gemcitabine and capecitabine in a chemotherapy regimen, as well as gemcitabine and capecitabine alone. In the study, which included 1,062 patients with locally advanced or metastatic PDAC, the GV1001 vaccine was not associated with an improved overall survival 129.​ Another phase III trial tested GV1001 versus combination gemcitabine and capecitabine in advanced PDAC patients with elevated serum eotaxin, with control patients receiving gemcitabine and capecitabine only. The OS of patients with advanced PDAC was significantly augmented by the trial group, as evidenced by the results 130.

### 5.3 CAR T-cells and oncolytic virus therapy

CAR T-cells and oncolytic virus therapy has shown promising results in the treatment of haematological malignancies 131, 132. A phase I trial (NCT01897415) verified the efficacy and safety of CAR-T therapy for immunotherapy-resistant mPDAC. Claudin-18 is a protein involved in the formation of tight junctions. Claudin 18.2 (CLDN 18.2) isoforms are expressed in 50%-70% of PDAC patients. A single-arm, open-label, first-in-human phase I pilot study (NCT03159819) evaluated the safety and efficacy of CLDN18.2-specific CAR T-cells in CLDN18.2-positive advanced pancreatic or gastric cancer. The results showed an ORR of 33.3%.

Genetically engineered viruses known as tumor oncolytic viruses (TOV) selectively infect and replicate in large numbers, lyse tumor cells without damaging normal cells, and can also provoke a powerful immune response that leads to tumor destruction. Pelareorep is an oncolytic reovirus that induces an anti-tumor immune response by activating the innate and adaptive immune systems. Pelareorep was found to be safe and effective in patients with advanced pancreatic cancer when combined with gemcitabine in a phase II study (NCT00998322) 133. No distinction in PFS was observed between the two groups when paclitaxel/carboplatin combined with pelareorep was tested in a randomised phase II trial (NCT01280058) compared to paclitaxel/carboplatin alone (4.9 months vs. 5.2 months) 134. In addition, the effects of pelareorep in combination with immune checkpoint inhibitors are being investigated. A phase Ib/II study (NCT02620423) explored the clinical effects of pelareorep in combination with chemotherapy and pembrolizumab in patients with mPDAC and showed superior safety and anti-tumor activity 135, 136. In November 2022, Oncolytics reported interim clinical data showing an ORR of 69% in a cohort of first-line advanced or mPDAC patients receiving a combination of pelarep, atezolizumab, and gemcitabine.​ This ORR is approximately three times the mean ORR reported in historical controlled trials evaluating GnP for pancreatic cancer. The clinical benefit rate was 85%, and as a result, the combination was granted Fast Track status by the FDA.

## 6. Conclusions and future directions

​Treatment of mPDAC is extremely difficult and challenging. Over the past few years, clinical trials for the treatment of mPDAC have proliferated, but with more hotspots and less consensus. In addition to systemic chemotherapy, local treatment (including resection, ablation and embolization) appears to improve survival (hepatic mPDAC 7.8-19 months, pulmonary mPDAC 22.8 -47 months), but more rigorous randomised controlled trials in mPDAC patients are needed to validate this 137. Researchers are trying to study the new molecular vulnerabilities of mPDAC. Recent advances in mPDAC include targeting metabolic pathways and identifying patients with germline and somatic alterations (e.g. patients with mutations in the BRCA1/2 and PALB2 genes).The POLO trial and FDA approval of olaparib as a maintenance therapy for mPDAC justify this direction. In addition, targeted TME and immune checkpoint inhibitor therapies are also therapeutic priorities for mPDAC and a broad portfolio of clinical immunological agents is yielding favorable results. As a result, future treatments of mPDAC will focus more on these hotspots.

## Figures and Tables

**Table 1:** Ongoing trials evaluating activity and efficacy of novel molecules acting on different targets in PC

	Target	Drug	Population	Additional therapy	Phase	Status	Clinical Trials.gov Identifier
**Metabolism**	TCA cycle	Devimistat	Metastatic Pancreatic Cancer	mFOLFIRINOX FOLFIRINOX	III	Completed	NCT03504423^30^
	Asparagine	L-Asparaginase	mPDAC	GemcitabineFOLFIRINOX	II	Completed	NCT02195180^38^
		Eryaspase	PDAC	Gemcitabine plus AbraxaneIrinotecan plus 5-FU plus leucovorin	III	Completed	NCT03665441^39^
			Locally Advanced PDACmPDAC	FOLFIRINOX	I	Active, not recruiting	NCT04292743 40
	MUC1	SM-88	Pancreatic Cancer	Capecitabine Gemcitabine 5-FU	II/III	Completed	NCT03512756^45^
			mPDAC	GnP mFOLFIRINOX Pamrevlumab combined with GnP Canakinumab and spartalizumab combined with GnP	III	Recruiting	NCT04229004
	Autophagy	HCQ	Untreated Resectable PDACBorderline Resectable PDACLocally Advanced PDAC	Paclitaxel protein boundGemcitabineCisplatin	II	Recruiting	NCT04669197
			mPDAC	Binimetinib	I	Recruiting	NCT04132505 57
			Metastatic Pancreatic CarcinomaUnresectable Pancreatic Carcinoma	Trametinib	I	Recruiting	NCT03825289^58^
			Advanced PDACmPDAC	Paricalcitol	II	Recruiting	NCT04524702
	ERK	Temuterkib	Pancreatic CancerAdvanced Cancer	Hydroxychloroquine Sulfate	II	Recruiting	NCT04386057
**DNA repair**	PARP (germline or somatic BRCA1/2 or PALB2 mutation)	Olaparib	mPDAC	Pembrolizumab	II	Recruiting	NCT04548752^66^
			Borderline Resectable PDACLocally Advanced PDACmPDACResectable PDAC	CobimetinibOnvansertibTemuterkib	I	Recruiting	NCT04005690 67
			mPDACPDAC	Cediranib Maleate	II	Active, not recruitin	NCT02498613^68^
		Rucaparib	mPDAC	FluorouracilIrinotecan SucrosofateLeucovorin Calcium	I/II	Active, not recruiting	NCT03337087
	ATM/ATR	Elimusertib	mPDACUnresectable PDAC	Irinotecan Hydrochloride	I	Recruiting	NCT04514497
		Ceralasertib	Locally Advanced Pancreatic CancerMetastatic Pancreatic Cancer	CeralasertibDurvalumab	II	Recruiting	NCT03682289
TME	CTGF	Pamrevlumab	Unresectable Pancreatic Carcinoma	GnP FOLFIRINOX	III	Active, not recruiting	NCT03941093
			mPDAC		III	Recruiting	NCT04229004
	FAK	Defactinib	Resectable PDAC	Pembrolizumab	II	Recruiting	NCT03727880 96
			Pancreatic Cancer		I/II	Recruiting	NCT02758587 97
	TGF-β	Galunisertib	Metastasis Pancreatic Cancer	Gemcitabine	I/II	Completed	NCT01373164 101
				Durvalumab	I	Completed	NCT02734160 102
	Vitamin	ATRA	PDAC	GnP	I	Completed	NCT03307148 107
					II	Not yet recruiting	NCT04241276
	CD40	Mitazalimab	mPDAC	mFOLFIRINOX	I/II	Recruiting	NCT04888312 138
		CDX-1140	PDAC	Pembrolizumab Chemotherapy CDX-301	I	Completed	NCT03329950
Immune system	CTLA4	Tremelimumab	Pancreatic Cancer	Gemcitabine	I	Completed	NCT00556023 117
		Ipilimumab	PDAC	Niraparib Nivolumab	I/II	Active, not recruiting	NCT03404960 121
	PD-1	Nivolumab	Pancreatic Cancer	Carboplatin GnP	I	Completed	NCT02309177 119, 139
	PD-L1	Durvalumab	PDAC	Tremelimumab GnP	II	Active, not recruiting	NCT02879318 120
			mPDAC	Tremelimumab	II	Completed	NCT02558894 122
	Vaccine	GVAX	mPDAC	Pembrolizumab Epacadostat Cyclophosphamide CRS-207	II	Active, not recruiting	NCT03006302
	CAR-T	CAR-T cells directed against CLD18	PDAC	None	I	Recruiting	NCT03159819 140
	TOV	Pelareorep	mPDAC	Gemcitabine	II	Completed	NCT00998322 133
				Carboplatin/Paclitaxel	II	Completed	NCT01280058 134
			PDAC	Pembrolizumab Chemotherapy	I	Completed	NCT02620423 135
